# Ocean Acidification Induces Changes in Virus–Host Relationships in Mediterranean Benthic Ecosystems

**DOI:** 10.3390/microorganisms9040769

**Published:** 2021-04-06

**Authors:** Michael Tangherlini, Cinzia Corinaldesi, Francesca Ape, Silvestro Greco, Teresa Romeo, Franco Andaloro, Roberto Danovaro

**Affiliations:** 1 Fano Marine Centre, Department of Research Infrastructures for Marine Biological Resources, Stazione Zoologica Anton Dohrn, Viale Adriatico 1-N, 61032 Fano, Italy; 2 Department of Materials, Environmental Sciences and Urban Planning, Polytechnic University of Marche, Via Brecce Bianche, 60131 Ancona, Italy; 3 Institute of Anthropic Impacts and Sustainability in Marine Environment-National Research Council (IAS-CNR), Lungomare Cristoforo Colombo n. 4521 (ex Complesso Roosevelt), Località Addaura, 90149 Palermo, Italy; francesca.ape@ias.cnr.it; 4 Department of Integrative Marine Ecology, Stazione Zoologica Anton Dohrn, Via Po 25c, 00198 Rome, Italy; silvestro.greco@szn.it; 5 Sicily Marine Centre, Department of Integrative Marine Ecology, Stazione Zoologica Anton Dohrn, Via dei Mille 46, 98057 Milazzo, Italy or teresa.romeo@isprambiente.it (T.R.); franco.andaloro@szn.it (F.A.); 6 National Institute for Environmental Protection and Research, ISPRA Via dei Mille 46, 98057 Milazzo, Italy; 7 Department of Life and Environmental Sciences, Polytechnic University of Marche, Via Brecce Bianche, 60131 Ancona, Italy; r.danovaro@staff.univpm.it

**Keywords:** ocean acidification, biodiversity, ecosystem functioning, prokaryotes, metabarcoding, viral impact

## Abstract

Acidified marine systems represent “natural laboratories”, which provide opportunities to investigate the impacts of ocean acidification on different living components, including microbes. Here, we compared the benthic microbial response in four naturally acidified sites within the Southern Tyrrhenian Sea characterized by different acidification sources (i.e., CO_2_ emissions at Ischia, mixed gases at Panarea and Basiluzzo and acidified freshwater from karst rocks at Presidiana) and pH values. We investigated prokaryotic abundance, activity and biodiversity, viral abundance and prokaryotic infections, along with the biochemical composition of the sediment organic matter. We found that, despite differences in local environmental dynamics, viral life strategies change in acidified conditions from mainly lytic to temperate lifestyles (e.g., chronic infection), also resulting in a lowered impact on prokaryotic communities, which shift towards (chemo)autotrophic assemblages, with lower organic matter consumption. Taken together, these results suggest that ocean acidification exerts a deep control on microbial benthic assemblages, with important feedbacks on ecosystem functioning.

## 1. Introduction

Over the last two hundred years, anthropogenic activities have led to the continuous increase in the concentration of atmospheric carbon dioxide (CO_2_), from 280 ppm in preindustrial times to a present-day level of ~400 ppm [1,2,3,4,5]. Atmospheric CO_2_ dissolves in seawater, leading to increased concentrations of inorganic carbon in the oceans with a corresponding reduction in mean surface seawater pH, a process called ocean acidification (OA) [4,5,6,7,8]. This process, coupled with ocean warming [9], poses significant threats to ocean life and ecosystem functioning on a global scale, such as mass mortalities and spread of pathogenic or invasive species, both in surface waters and in the deep ocean. These changes, influencing carbonate chemistry, have been shown to have different impacts on marine life, especially on calcifying species such as corals [4,10], seagrasses and macroalgae [11,12,13], but also meiobenthic taxa, which are directly responsible for the energy transfer to high trophic levels [7]. Microbial diversity is also expected to be influenced by OA, as found by previous investigations [5,12]. Those studies indeed showed that volcanic CO_2_ venting can provide important evidence on the role and impact of pH reduction on prokaryotic-mediated activities (e.g., organic matter degradation, nitrogen fixation and nitrification) [5,7,14] and have an impact on prokaryotic diversity (in terms of both alpha-diversity and assemblage structure) [5,6].

However, the results provided by those studies might be of limited support to research on the effects of ocean acidification, as either: (i) they mostly focus on the water column, lacking information on benthic environments, which provide a wide array of ecosystem services [15,16,17], (ii) the results were diverging and hard to reconcile, suggesting that the high variability in responses and shifts in prokaryotic assemblage structure and functional responses might be related to the environmental complexity of naturally acidified sites [15,18] and (iii) they were based on laboratory experiments, which do not reflect the real ecosystem dynamics underlying natural assemblages or include few sampling points [6,8,16,19].

In addition, the effects of acidification on prokaryotes can also alter the interactions they have with viruses [20,21]. By manipulating their hosts (through killing or metabolic reprogramming), viruses exert a strong influence on marine food webs’ functioning [2,22,23,24], thus converting an important fraction of prokaryotic C production into organic detritus, which can in turn be used by other organisms [25,26]. Due to their global abundance and relevance, any potential ecosystem change acting (directly or indirectly) on viruses might thus have (currently unpredictable) feedbacks on global ecosystem dynamics [2]. Indeed, previous studies found that virus–host interactions (e.g., burst size, lytic-lysogenic cycle switching) are influenced by physical-chemical characteristics of their environments (e.g., salinity, trophic status, temperature) [2], for example, seasonal dynamics in viral abundance are regulated by their hosts’ productivity in surface waters, while nutrient limitation appears to be linked to a prevalence of lysogeny [2]. However, very few studies have been focused on detailing the impact of ocean acidification on virus–host interactions [27,28,29], viral survival/replication [2,30] and host metabolism stimulation by C release [26]. Most of such studies highlighted that the complex network of virus–host relationships can be heavily influenced by global changes [27,28,29,30], altering organic matter remineralization and the overall ecosystem functioning. Thus, understanding the response of prokaryotes, viruses and their interactions to ocean acidification could be of paramount importance to explain the variable response capability for deeply influencing geochemical cycles [2,22,26,31,32].

To this extent, “natural laboratories” (i.e., sites or areas affected by acidification by natural CO_2_ inputs) [5,33,34] provide shallow depth “windows” into long-term effects of acidification on marine benthic communities and its impact on key biological processes. In particular, several environments within the Southern Tyrrhenian Sea (e.g., the island of Ischia and the Aeolian Arc) [34,35] offer this opportunity and have been recognized as fundamental sites for this investigation. Thus, “natural laboratories” represent the best chance to study these processes and predict future trends in marine ecology.

For this purpose, in the present work, we investigated three marine habitats within the Southern Tyrrhenian Sea, which hosts a number of naturally acidified shallow sites offering invaluable insights into the long-term OA effects on organisms and biogeochemical cycles. These three sites are naturally acidified but differ in terms of pH values and CO_2_ sources. We investigated the effects of OA on microbial processes in the benthic ecosystem, analyzing prokaryotic abundance and extracellular enzymatic activities, viral abundance and impact on prokaryotes and the prokaryotic assemblage. The results of the present study can provide novel insights on the effects of OA on these microbial components and their potential cascading effects on ocean functioning.

## 2. Materials and Methods

### 2.1. Sampling Areas

The Southern Tyrrhenian Sea hosts a number of naturally acidified sites, which can offer invaluable insights into the OA effects of long-term acidification on organisms and biogeochemical cycles [33,34] as these shallow venting systems represent windows to the future of the oceans. Here, we investigated 4 sites from 3 different naturally acidified systems from the Southern Tyrrhenian Sea: Ischia, Presidiana and the Panarea island (Table 1 and Supplementary Figure S1).

The area of Ischia is represented by volcanic CO_2_ vent systems in the Tyrrhenian Sea (Naples, Italy), where submarine CO_2_ vents occur at ca. 3 m depth, lowering the pH values of the overlying water from >8.0 to <7. As reported by previous studies [35], seawater receives gases largely represented by CO_2_ (90.1–95.3%).

The area of Presidiana is represented by a bay in the Tyrrhenian Sea (Sicily, Italy), characterized by natural acidification of surface waters (reductions in average pH values of 0.2–0.5 units) comparable to the predicted 2100 scenario [3,36] due to the presence of a spring originating from karst rocks (Madonie system), in which water is characterized by high values of pCO_2_ (from 500 to 3600 μatm) and HCO_3_^-^ (on average 3700 µmol kg^−1^), high alkalinity (on average ca. 3900 µmol kg^−1^) and salinity of ca. 22, which extends across the bay.

The sites of Panarea (Basiluzzo and Secca dei Pesci, Panarea Island) are characterized by the release of CO_2_ (from 92% to 95%) and minimal concentrations of H_2_S, N_2_ and O_2_ [37], and feature a range of complex structures of different venting areas [38].

### 2.2. Sediment Sampling Strategy and Storage

Sampling was carried out at Ischia and Presidiana sites by scuba diving. Samples from the Ischia area were collected at 3 sites: 2 around the vents (defined as N2 and S3; 3–5 m depth) and 1 considered as control (defined as N1). Samples collected in Summer (October 2011) were marked with an “a” and samples collected in Winter (January 2013) were marked with a “b”. Samples from the Presidiana area were collected at 2 sites (at ca. 1 m depth): 1 close to the spring source and 1 along the flow (defined as P1 and P4, respectively), and at 1 control site (defined as P7) located at the northern side of the bay. Samples collected in Summer (June 2010) were marked with an “a” and samples collected in Winter (February 2011) were marked with a “b”. At both Ischia and Presidiana areas, pH values were measured in situ both in surface waters and at the sediment–water interface, with a YSI Total Dissolved Solids (TDS) conductivity meter. Sediment pH was also determined using a Crison pH electrode/meter calibrated with NBS buffers (accuracy ± 0.005).

Sampling in the Panarea area was carried out during the ISPRA “ORBS PANA_15” scientific cruise on board of the *R/V Astrea* by means of a Van Veen grab. Two different sub-areas were explored: one off the South-western coast of the islet of Basiluzzo (defined as CB31, CB32, CB33 and CB34) and one at the Southeastern sector of the Panarea Volcanic Complex, in the shallow region (ca. 40 m of depth) known as “Secca dei Pesci” (defined as SP1, SP2, SP3 and SP4). The chosen areas were first characterized by means of multi-beam prospection, which allowed to identify prospective venting sites within the two sub-areas [38]; within the Basiluzzo area, a benthic chamber was deployed for 6 h along 3 different sites within a close depth range (74–81 m of depth) characterized by potential venting activity to carry out measurements of temperature, dissolved inorganic carbon (DIC), metals and H^+^ over time. Low-flux sites were utilized as “control” samples. In the Secca dei Pesci sub-area, pH and T were measured through a CTD cast along the water column, which allowed to select one venting and one non-venting sampling site within the sub-area, and 2 additional samples were collected far from the SP sub-area at comparable depths (range: 36–41 m of depth).

Sediment samples were collected in three independent replicates. The surface (0–1 cm) layer of each sediment core was utilized for the microbiological analyses. Once retrieved, cores were kept at in situ temperature until further subsampling in the laboratory. At Panarea sites, 3 independent deployments were performed at each sampling site, sediments were collected using plexiglass cores and subsequently sliced in layers (0–1, 1–3, 3–5, 5–10, 10–15 cm) and the top first cm of each core was used for all the analyses.

For the determination of prokaryotic abundance, ca. 1 g of surface sediment was diluted immediately after sampling with 1 mL of 0.2 μm pre-filtered seawater (sediment:water volume 1:1), fixed with formalin (final concentration 2%) and stored at 4 °C until processing (within two weeks) [39]. For the determination of viral abundance, samples were stored at −20 °C until the analysis in laboratory (within two weeks). For the analysis of viral production, sediment samples were stored at −20 °C at different time ranges (immediately after dilution, after 1.5 h, after 3 h, after 6 h, after 12 h). For each time range, ca. 1 g of surface sediment was diluted immediately with 1 mL (1:1) of virus-free seawater pre-filtered onto 0.02 μm membranes and incubated at in situ temperature in the dark until freezing [26].

For the analysis of extracellular enzymatic activity, ca. 1 g of sediment from the top 1 cm was transferred immediately after sampling in a sterile tube (one for each substrate) supplemented with 1 mL (1:1) of seawater prefiltered onto 0.2 μm membranes (Anodisc, diameter 25 mm) and stored at −20 °C. For the determination of organic matter composition and DNA extraction, sediment samples were split in separate petri dishes and sterile tubes and stored at −20 °C. For the analysis of prokaryotic heterotrophic carbon production and viral production, aliquots of sediment from the top first cm of each core were immediately analyzed as described below.

### 2.3. Biochemical Composition of Sediment Organic Matter

Protein, carbohydrate and lipid were determined spectrophotometrically, following the protocols detailed in Reference [39], and their sedimentary contents (as mg of proteins, carbohydrates and lipids per gram of dry sediment) expressed as bovine serum albumin, glucose and tripalmitine equivalents, respectively. Carbohydrate, protein and lipid sedimentary contents were converted into carbon equivalents using the conversion factors of 0.40, 0.49 and 0.75 μg C μg^−1^ respectively, and their sum defined as biopolymeric carbon (BPC) [40]. Analyses were carried out on 3 replicates for each site.

### 2.4. Viral Abundance, Production and Viral-Induced Prokaryotic Mortality

For the determination of viral abundance and viral production, stored sediment samples were treated as in Reference [39]. Briefly, surface sediment slurries and slurries of each time stop for viral production were treated with tetrasodium pyrophosphate (100 mmol L^−1^ final concentration) and ultrasound treatment (1 min of treatment followed by 30 s of manual shaking, repeated 3 times), before undergoing centrifugation (800× *g*, 1 min) to pellet the sediment particles. The supernatant was diluted 200× with virus-free seawater. Diluted samples were supplemented with DNase I from bovine pancreas (10 U mL^−1^ final concentration) and incubated for 20 min at room temperature in the dark. Then, they were filtered on 0.02 μm pore size 25 mm aluminum oxide filter membranes (Anodisc) and stained with 100 μL of SYBR Gold (10,000× stock solution diluted 1:5000 in Tris-EDTA buffer). Filters were incubated in the dark for 20 min, washed 3 times with 3 mL of virus-free seawater and then mounted on glass microscope slides with 20 μL of antifade solution (5% phosphate-buffered saline pH 7.8; 5% glycerol, 0.5% ascorbic acid). Viral abundance count was carried out by epifluorescence microscopy (magnification 1000×), analyzing at least 20 optical fields or 200 viruses. Microscope slides were stored at −20 °C. Data were normalized to sediment dry weight. The analysis was carried out on 3 replicates for each site. Net viral production was determined as the maximum increment of viral abundance per g of dry sediment per hour.

The burst size (i.e., the average number of virus released by a single prokaryotic cell due to viral infection) was assumed to be of 45 virus cell^−1^ [23]. The C released by the viral shunt was calculated by converting the number of killed prokaryotes into C content using 20 fgC per cell as a conversion factor [27]. The number of killed prokaryotes (KP) was calculated as Viral Production/Burst Size and Viral-Induced Prokaryotic Mortality (VIPM) was calculated as KP × 100/Prokaryotic Abundance [26].

### 2.5. Prokaryotic Abundance

For the estimation of prokaryotic abundance, stored sediment samples were treated as in Reference [39]. Surface sediment slurries were treated with tetrasodium pyrophosphate (100 mmol L^−1^ final concentration) and ultrasound treatment (1 min of treatment followed by 30 s of manual shaking, repeated 3 times), before undergoing centrifugation (800× *g*, 1 min) to pellet the sediment particles. The supernatant was diluted with virus-free seawater. Diluted samples were filtered on 0.2 μm pore size 25 mm filter membranes and stained with 100 μL of SYBR Green I (10,000× stock solution diluted 1:20 in Tris-EDTA buffer). Filters were incubated in the dark for 20 min, washed 3 times with 3 mL of virus-free seawater and then mounted on glass microscope slides with 20 μL of antifade solution (5% phosphate-buffered saline pH 7.8; 5% glycerol, 0.5% ascorbic acid). Prokaryotic abundance count was carried out by epifluorescence microscopy (magnification 1000×) analyzing at least 20 optical fields or 200 prokaryotic cells. Microscope slides were stored at −20 °C. Data were normalized to sediment dry weight. The analysis was carried out on 3 replicates for each site.

### 2.6. Extracellular Enzymatic Activities

For the analysis of extracellular enzymatic activities, samples were supplemented with 2.9 mL of seawater prefiltered onto 0.2 μm membranes for a final total volume of 4.9 mL. Extracellular enzymatic activities (aminopeptidase, β-glucosidase and alkaline phosphatase) were determined as described in Reference [39]. Analyses were carried out by adding 100 μL of Leu-MCA and Glu-MUF and 200 μL of MUF-P fluorometric analysis at fixed wavelength (Leu-MCA: 380 nm excitation and 440 nm emission, Glu-MUF/MUF-P: 365 nm excitation and 455 nm emission) at low and medium level of sensibility in t0 and in t1 (substrate incubations were performed in the dark at in situ temperature for 1 h). The increase of fluorescence units during incubation was converted into activity by comparison with standard curves of 7-amino-4-methylcoumarin for Leu-MCA and of 4-methylumbelliferone for both Glu-MUF and MUF-P. Enzymatic activities were expressed as nmol of substrate hydrolyzed L^−1^ h^−1^. Analyses were conducted on 3 replicates for each site. The amount of the artificial fluorogenic substrate hydrolyzed by proteases and glucosidases were converted respectively into protein and carbohydrate degradation rates using 72 µg of C per micromole of substrate hydrolyzed and expressed as µg of C degraded g^−1^ h^−1^.

### 2.7. Prokaryotic Diversity

For the analysis of prokaryotic diversity, approximately 5 mL of frozen sediment samples were used for the DNA extraction. Total DNA was extracted using a Power Max Soil DNA Isolation Kit (Mo-Bio Lab, Carlsbad, CA, USA) with the modifications provided in Reference [39] for the removal of extracellular DNA. The DNA extracted from sediment samples from the Panarea island was sequenced on an Illumina MiSeq sequencer using the V3 technology (2 × 300 bp), whereas those from the Ischia and Presidiana sites were sequenced on a Roche Life Sciences 454 Titanium FLX platform. All libraries were prepared, independently from the sequencing platform, with two different sets of primers, one targeting the Archaeal V4–6 region and one targeting the Bacterial V4 region [41].

Raw paired-end sequences from the two different technologies were imported into the QIIME2 pipeline [42]. Within QIIME2, leftover primer sequences were first removed from all sequencing runs through the cutadapt program [42,43]. The DADA2 plugin for the QIIME2 pipeline [42] was then used to separately denoise 454 sequences (through the *denoise-pyro* command) and the forward-facing only files of the MiSeq sequencing run (through the *denoise-single* command), truncating sequences in both sequencing runs at 250 bp.

Representative sequences (ASVs) and ASV tables from both denoising runs for both Archaea and Bacteria were then merged within QIIME2 (with the *--p-overlap-method* set to “sum” to sum relative abundance of identical ASVs in the two feature tables). A first round of taxonomic affiliation of ASV was carried out on a subset of the SILVA v.138 database [44], trimmed through the *extract-reads* procedure on the region amplified by the chosen primer sets, through the *classify-consensus-vsearch* classifier, with standard parameters [45], and ASVs matching mitochondrial and chloroplast sequences were removed. The remaining ASVs were then placed on a reference tree using the *q2-fragment-insertion* plugin within QIIME2, which supports the SEPP algorithm [46], to remove possibly spurious ASVs generated by the analysis and to better compare results from different sequencing platforms. Finally, a second round of taxonomic affiliation was carried out on the same database subset to also remove eukaryotic and unaffiliated sequences.

ASVs were aligned and the alignment was used to create a rooted tree within QIIME2 using the implemented MAFFT and FastTree tools. Phylogenetic data obtained in this way was then utilized for the computation of alpha-diversity indices (i.e., ASV richness, Pielou’s index, Shannon index and Faith’s phylogenetic diversity) after rarefying at 20,000 sequences to allow the comparison of all samples despite differences in sequencing depths [47].

### 2.8. Statistical Analyses

Statistical analyses on sediment biochemical characteristics, prokaryotic abundance and activity, viral abundance, production and impact on prokaryotes were carried out within R [48]. The *factoextra* package [49] was used to carry out Principal Component Analysis (PCA) on the biological variables considered (i.e., prokaryotic abundance, viral abundance, viral production, biopolymeric C concentration, chlorophyll pigments equivalents and organic matter degradation rate), to cluster samples and identify the contribution of variables to PCA axes. Subsequently, comparisons were carried out between values of each variable considered between clusters using the *GGPubr* package [50], which performed Wilcoxon tests between samples along with False Discovery Rate *p*-value adjustments. Adjusted significant differences were reported and marked on the associated bar plots. The same statistical analyses were performed on alpha-diversity results.

Statistical analyses were performed to evaluate significant differences in prokaryotic assemblages of samples from each cluster considering the biases introduced by massive sequencing technologies (e.g., sparsity and compositionality) [51]. To circumvent these biases, we utilized the multinomial regression approach provided by the Songbird QIIME2 plugin [52] using samples from the C1 cluster as a reference to infer differentially abundant prokaryotic families in target clusters C2 and C3. The top 20% of the differentially abundant families at both numerator (i.e., more abundant in samples from target clusters) and denominator (i.e., more abundant in samples from the reference cluster) were then selected through the Qurro tool [53]. At the same time, the main ASV abundance table was collapsed at the family level and then treated using a centered log-ratio transformation (CLR) [51], with the addition of +1 as a pseudo-count to treat zero-count observations. Differentially abundant families were extracted from the table and used as an input for a linear regression analysis carried out with the *GGPubr* package [50], to highlight correlations (using the Pearson’s test) between prokaryotic taxa and biotic variables, and only correlations with R > 0.6 and *p* < 0.05 were kept.

## 3. Results

### 3.1. Environmental Characteristics

Sampling sites within the Ischia area were characterized by sandy sediments, whereas those within the Presidiana area were characterized by a coarser sediment texture. Sediments from both Panarea sampling sites ranged from coarse sand (at the SP sub-area at all non-venting sites) to a thick reddish crust covering three out of four sites at the Basiluzzo area, either inside or outside the venting site.

The analysis of pH values showed a seasonal variation at both Ischia and Presidiana sampling areas. At Ischia, pH values ranged from 8.01 (controls) to 6.39 (acidified sites) in winter and from 7.94 to 6.01 in summer, whereas at Presidiana, they ranged from 7.84 to 8.04 in winter and from 7.97 to 8.16 in summer (Supplementary Figure S1). At SP, values ranged from 5.8 (for the venting site SP1) to 7.8 (for both control sites SP3 and SP4), whereas at Basiluzzo, they ranged from 8.07 to 8.22 for non-venting sites and 6.07 for the venting site CB33 (Supplementary Figure S2).

### 3.2. Effects of Acidification on Biological Variables

**Figure 1:**
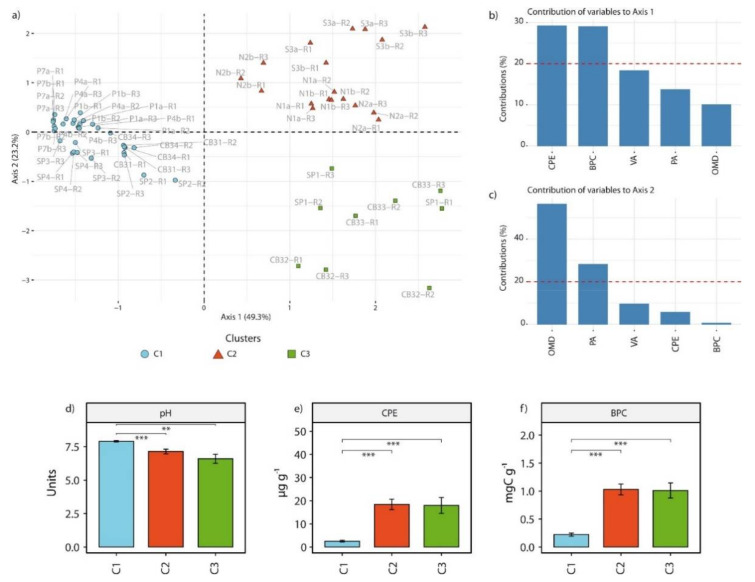
Results of k-means clustering and PCA carried out on biotic variables analyzed across all sites. (**a**) PCA plot showing the distribution of samples in a two-dimensional space, with the amount of variance explained by each axis and affiliation of sampling points to each cluster. (**b**) Contribution of the 5 variables analyzed to the variance explained by axis 1, (**c**) contribution of the 5 variables analyzed to the variance explained by axis 2, (**d**–**f**) histograms showing the average values for pH, chlorophyll pigments equivalents and biopolymeric C concentrations among the 3 clusters identified, with asterisks displaying significant differences between clusters (** for *p*-values < 0.01, *** for *p*-values < 0.001) and error bars computed on the standard error for each cluster. PA: prokaryotic abundances (cell g^−1^); VA: viral abundances (viruses g^−1^); BPC: biopolymeric C concentration (mgC g^−1^); CPE: chlorophyll pigment equivalents (µg g^−1^); OMT: organic matter turnover (µgC g^−1^ h^−1^).

The combination of K-means clustering and PCA carried out on the main biological variables investigated showed that samples could be partitioned in 3 clusters according mainly to biopolymeric C concentration (contributing up to 29% to the first axis), chlorophyll pigment equivalents (contributing up to 29% to the first axis), organic matter degradation rates (contributing up to 56% to the second axis) and prokaryotic abundance (contributing up to 28% to the second axis; Figure 1a–c): (i) one containing samples from Basiluzzo, SP and Presidiana, characterized by significantly higher pH values (on average, 7.90 ± 0.29; Wilcoxon test, *p*-value < 0.01) and lower BPC and CPE values (on average, 0.22 ± 0.16 mgC g^−1^ and 2.54 ± 2.33 µg g^−1^, respectively; Wilcoxon test, *p*-value < 0.001; Figure 1d–f) than in the other clusters, thus hereafter defined as “C1”. (ii) One containing only samples from Ischia, with intermediate pH values (on average, 7.14 ± 0.77), thus hereafter defined as “C2”, and (iii) one only containing samples from Basiluzzo and SP characterized by bubbling and surface crusts, thus hereafter defined as “C3” (Figure 1a). 

### 3.3. Prokaryotic Abundance and Activity

**Figure 2:**
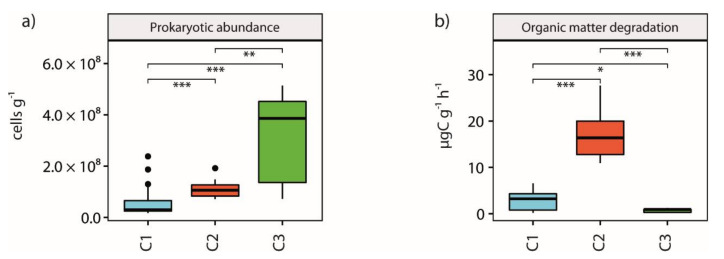
Impact of acidification on prokaryotic abundance and dynamics. Histograms showing the average values for prokaryotic abundances (**a**) and organic carbon degradation rates (**b**) among the 3 clusters identified, with asterisks displaying significant differences between clusters (* for *p*-values < 0.05, ** for *p*-values < 0.01, *** for *p*-values < 0.001) and error bars computed on the standard error for each cluster.

Analysis showed that prokaryotic abundances were always significantly (*p*-value < 0.01) higher in C2 and C3 sites than in C1 ones (5.23 ± 5.08 × 10^7^ viruses g^−1^, on average, for the C1 sites vs. 1.10 ± 0.32 × 10^8^ and 3.13 ± 1.76 × 10^8^ viruses g^−1^, on average, for C2 and C3 sites respectively, Figure 2a). A significantly (*p*-value < 0.001) higher organic C degradation rate was found in C2 sites than in others (17.38 ± 5.15 µg C g^−1^ h^−1^, on average, vs. 2.84 ± 2.01 µg C g^−1^ h^−1^ and 0.74 ± 0.39 µgC g^−1^ h^−1^, on average, for C1 and C3 sites respectively, Figure 2b).

### 3.4. Viral Abundance and Production and Viral Impact on Prokaryotes

**Figure 3:**
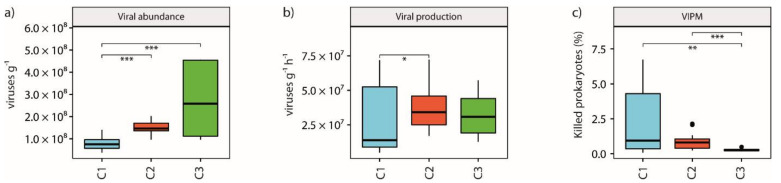
Impact of acidification on viral abundance and dynamics. Histograms showing the average values for viral abundances (**a**), viral production (**b**) and viral-induced prokaryotic mortality (VIPM; **c**) among the 3 clusters identified, with asterisks displaying significant differences between clusters (* for *p*-values < 0.05, ** for *p*-values < 0.01, *** for *p*-values < 0.001) and error bars computed on the standard error for each cluster.

Analyses showed that viral abundance was significantly (*p*-value < 0.001) lower in C1 than in C2 and C3 clusters (8.02 ± 2.87 × 10^7^ viruses g^−1^, on average, vs. 1.52 ± 0.27 × 10^8^ and 2.67 ± 1.54 × 10^8^ viruses g^−1^, on average, for C2 and C3 sites respectively, Figure 3a). Slight but significant (*p*-value < 0.05) differences were found between C1 and C2 sites (2.60 ± 2.23 × 10^7^ viruses g^−1^ h^−1^, on average, vs. 3.72 ± 1.63 × 10^7^ viruses g^−1^ h^−1^, on average respectively, Figure 3b). Viral-induced prokaryotic mortality (VIPM) was however significantly (*p*-value < 0.01) lower between C3 and C2 sites (0.27 ± 0.1%, on average, vs. 0.86 ± 0.55%, on average, respectively, Figure 3c) and between C3 and C1 sites (0.27 ± 0.1%, on average, vs. 2.21 ± 2.26%, on average, respectively, Figure 3c).

### 3.5. Microbial Assemblage Diversity and Structure

**Figure 4:**
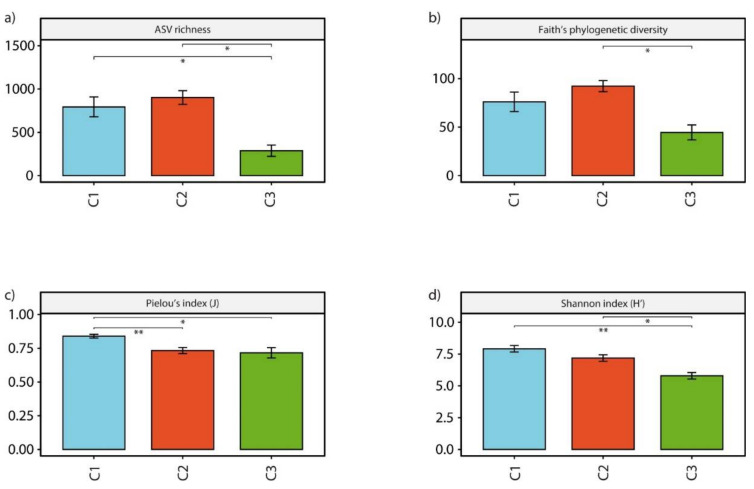
Impact of acidification on prokaryotic α-diversity. Histograms showing the average values for ASV richness (**a**), Faith’s phylogenetic diversity (**b**), Pielou’s index (**c**) and Shannon index (**d**) among the 3 clusters identified, with asterisks displaying significant differences between clusters (* for *p*-values < 0.05, ** for *p*-values < 0.01) and error bars computed on the standard error for each cluster.

Analysis of alpha-diversity indices for the prokaryotic assemblages showed that ASV richness values were significantly (*p*-value < 0.05) lower in C3 sites than among the others (287.67 ± 112.19, on average, vs. 902.00 ± 194.38 and 793.64 ± 380.54, on average, for C2 and C1 sites respectively, Figure 4a). Similarly, phylogenetic diversity was significantly (*p*-value < 0.05) lower in C3 sites than in C2 sites (44.56 ± 13.31, on average, vs. 92.22 ± 14.02, Figure 4b). Conversely, both Pielou’s and Shannon indices showed significantly (*p*-value < 0.05) higher values in C1 sites than in C2 and C3 ones (0.84 ± 0.05, on average, vs. 0.73 ± 0.06 and 0.72 ± 0.06, on average, for C2 and C3 sites respectively, and 7.92 ± 0.85, on average, vs. 7.19 ± 0.62 and 5.79 ± 0.44, on average, for C2 and C3 sites respectively, Figure 4b,c).

**Figure 5:**
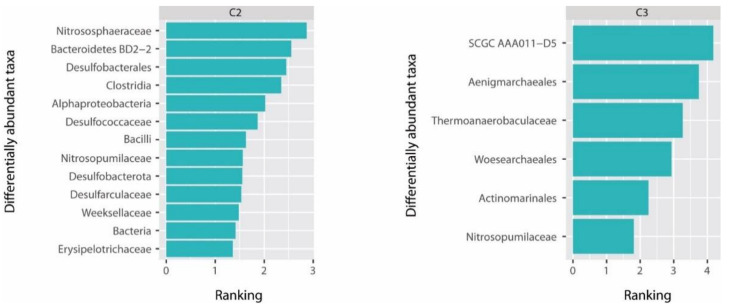
Results of Songbird analysis including top 20% of differentially abundant taxa at family level associated with samples from clusters C2 and C3 with samples from cluster C1 as reference.

Analysis of differential abundances showed that several taxa, such as families *Nitrososphaeraceae* and BD2-2, unclassified Desulfobacterales, Clostridia and Alphaproteobacteria were more abundant in samples from cluster C2 (differential ranking > 2, up to 2.87, Figure 5), whereas families SCGC_AAA011-D5, *Thermoanaerobaculaceae* and unclassified Woesearcheales and Aenigmarchaeales and uncultured Actinomarinales were strongly associated with samples from cluster C3 (differential ranking > 2, up to 4.18, Figure 5). Families more abundant in samples from cluster C1 included archaeal Lokiarchaea and Bathyarchaeia and bacterial *Staphylococcaceae*, *Vicinamibacteraceae*, *Microbacteriaceae* and *Xenococcaceae* (Figure 5, Supplementary Figure S3).

**Figure 6:**
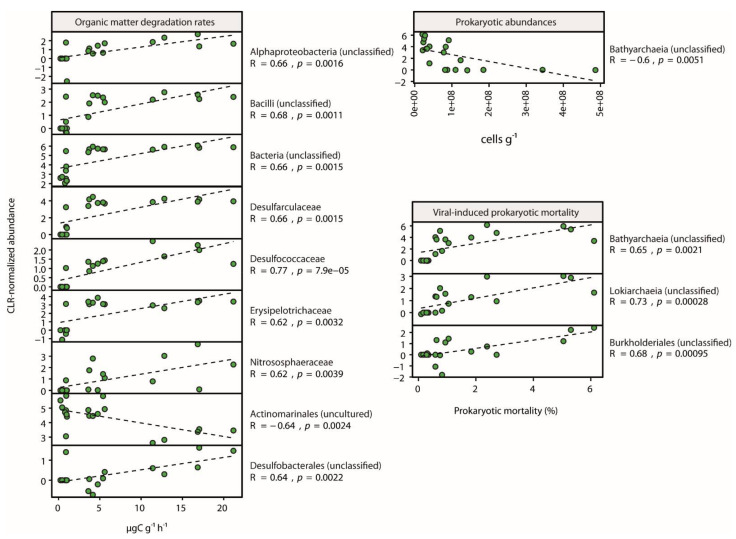
Linear regression analysis on CLR-normalized abundances of differentially abundant prokaryotic taxa at the family level, only considering taxa with R > 0.6 and *p*-values < 0.05.

Linear regression analyses on differentially abundant taxa at the family level showed that the normalized abundances of up to 8 different taxa (including both Archaea and Bacteria) were significantly (*p*-value <0.01) and positively correlated with organic matter degradation rates, with only uncultured Actinomarinales responding negatively (Figure 6). Interestingly, unclassified Bathyarchaeia were significantly (*p*-value < 0.01) negatively correlated with prokaryotic abundances but positively (along with unclassified Lokiarchaeia and Burkholderiales) with VIPM values (Figure 6).

## 4. Discussion

The importance of naturally acidified marine ecosystems as “natural laboratories” to study the impact of OA on key biological processes has been increasingly recognized over time [54], involving all levels of biological organization, from viruses to macrofauna and seagrasses [8,10,11,55].

At the same time, the different sources of venting (e.g., presence of gases in addition to CO_2_, such as H_2_S) and environmental conditions (e.g., different trophic regimes, environmental stability, temperature, seabed composition) can determine differences in the potential response that can overlap with the environmental and trophic conditions [5,54,55,56,57,58,59].

In order to clarify and disentangle the effects of different variables on the microbial components, we compared the impacts of acidification from multiple sources in four “natural laboratories” within the Southern Tyrrhenian Sea. The sites are characterized by clear differences in terms of source of acidification (gas emissions at both Ischia and Panarea, freshwater from a karst system at Presidiana), gas composition (e.g., gases largely represented by CO_2_ at Ischia and CO_2_ with traces of H_2_S at Panarea) [35,36,37] and seabed composition (sandy sediments at Ischia, coarse and crustose deposits at Panarea) [38], which reflect the high variability in environmental conditions that can be found in “natural laboratories”. We found that, despite their structural differences and different sampling areas, samples collected from the four areas could be assigned to 3 different clusters: non-acidified samples (either from the Panarea—Secca dei Pesci or Presidiana areas) were included in cluster C1, samples from the area of Ischia (pH values < 8.1 [35]) were included in cluster C2 and samples from the two highly acidified areas (Panarea—Secca dei Pesci and Basiluzzo areas [39]) were included in cluster C3.

Here, we show that, in acidified conditions, viral infections (expressed as the percentage of prokaryotes killed by viruses, VIPM) were significantly lower (within cluster C3) than in the controls (clusters C1 and C2), indicating that low pH values can reduce the top-down control on prokaryotic biomass [23]. Interestingly, both prokaryotic and viral abundances were maintained relatively high in the clusters affected by acidification (C2 and C3). High viral abundance values at high host densities suggest that the life strategy utilized by viruses in such clusters is more likely to be temperate (i.e., not inducing cell lysis), than lytic [21,23,60,61,62]. Indeed, a temperate (possibly chronic) life strategy would reduce the impact of viral mortality on prokaryotes (thus explaining the high host abundance and low VIPM values in clusters C2 and C3) [27], ensuring at the same time a continuous viral replication. This finding is not only supported by mathematical models, which suggest that temperate and chronic viral infections can coexist when host abundances are high [62], but also by recent studies which revealed that widespread heterotrophic marine bacteria are influenced by chronic bacteriophage infections [63]. We therefore hypothesize that chronic infection might represent an important life strategy for prokaryotic viruses in acidified environments.

The impact of acidification on the prokaryotic fraction can be observed both in terms of prokaryotic activity (through organic matter-degrading extracellular enzymes) and diversity. In particular, samples from cluster C2 were enriched in both nitrifying Archaea (belonging to families *Nitrososphaeraceae* and *Nitrosopumilaceae*) [64,65] and sulfate-reducing prokaryotes (belonging to the families *Desulfococcaceae* and *Desulfarculaceae*, to order Desulfobacterales and phylum Desulfobacterota), indicating that acidification might induce changes in key geochemical cycles (such as those of nitrogen and sulfur) [2]. In addition, in cluster C3 (comprising hydrothermal vent-influenced samples from the Panarea area), high prokaryotic abundances were coupled with low rates of organic matter degradation, suggesting that heterotrophic C consumption might not be the primary metabolic pathway in these samples. We can thus hypothesize that (chemo)autotrophic C production could be responsible for sustaining the high prokaryotic abundances in this cluster. This hypothesis is not only consistent with previous studies (which showed that high pH conditions lead to lower rates of heterotrophic C production and higher amounts of biopolymeric C within sediments, as also found in our study) [66], but also with taxonomic analysis, which shows that samples from cluster C3 are associated with thermophilic and extreme environments (*Thermoanaerobaculaceae*) [67], together with potentially syntrophic Archaea (Woesearchaeales, Aenigmarchaeales) [68], revealing a complex network of interactions in a less diversified environment (as indicated by alpha-diversity analyses).

Finally, regression analyses conducted in the present study showed the presence of significant relationships: (i) several sulfate-reducing taxa (i.e., *Desulfarculaceae*, *Desulfurococcaceae* and Desulfobacterales) were positively correlated with organic matter degradation rates, suggesting the role/contribution of this group to C degradation (which is supported for some of the cultured strains of these groups) [67], and (ii) Bathyarchaeia and Lokiarchaeia were positively correlated with viral induced prokaryotic mortality, suggesting that either viral infections can preferentially impact other taxa thus reducing the abundance of their competitors, or that these taxa are efficient in utilizing organic carbon released by viral infections. We thus provide, for the first time, insights on the importance of such widespread taxa in acidified ecosystems and their potential relationships with viruses, which are still uncharacterized.

## 5. Conclusions

Since the sources of acidification, pH values, venting intensity and environmental conditions are different in the three investigated areas, it is difficult to draw general rules on the impact of the progressive ocean acidification at a global scale. However, the results of the present study suggest that acidified conditions can have significant effects on benthic microbial components. These effects can be summarized as: (a) shift in viral life strategies from mainly lytic in non-acidified sites to co-existing temperate strategies (i.e., chronic infection and lysogenic cycle) in acidified sites, (b) increased prokaryotic abundances (also due to lower viral impact) at lower pH values, (c) development of chemoautotrophic communities at significantly lower pH values and (d) changes in ecosystem functioning with lower organic matter degradation rates at acidified sites.

## Figures and Tables

**Table 1:** Sampling site coordinates and area.

Site	Area	Latitude (N)	Longitude (E)
P1	Presidiana	38.038236°	14.032400°
P4	Presidiana	38.037050°	14.032194°
P7	Presidiana	38.041211°	14.025622°
N2	Ischia	40.732217°	13.964150°
N3	Ischia	40.732167°	13.963733°
S3	Ischia	40.730883°	13.963017°
SP1	Panarea (Secca dei Pesci)	38.594667°	15.115617°
SP2	Panarea (Secca dei Pesci)	38.593883°	15.111633°
SP3	Panarea (Secca dei Pesci)	38.628250°	15.048667°
SP4	Panarea (Secca dei Pesci)	38.637117°	15.050883°
CB31	Panarea (Basiluzzo)	38.650883°	15.102550°
CB32	Panarea (Basiluzzo)	38.647550°	15.108500°
CB33	Panarea (Basiluzzo)	38.650967°	15.093333°
CB34	Panarea (Basiluzzo)	38.655950°	15.088150°

## Data Availability

Sequence data is available within the NCBI BioProject PRJNA719925.

## Supplementary Materials

The following are available online at https://www.mdpi.com/article/10.3390/microorganisms9040769/s1. Supplementary Figures S1–S3 are available online together with the manuscript.
