# Supplementary material for: Ocean Acidification Induces Changes in Virus–Host Relationships in Mediterranean Benthic Ecosystems
Source: Microorganisms. 2021 Apr 6;9(4):769. doi: 10.3390/microorganisms9040769 (PMC8067541; doi:10.3390/microorganisms9040769)
Supplement: Supplementary file 1 [file microorganisms-09-00769-s001.pdf]

Article

Ocean acidification induces changes in virus-host relationships in Mediterranean benthic ecosystems

Michael Tangherlini[1*], Cinzia Corinaldesi[2], Francesca Ape[3], Silvestro Greco[4], Teresa Romeo[5,6], Franco Andaloro[5] and Roberto Danovaro[7]

1 Department of Research Infrastructures for marine biological resources, Stazione Zoologica Anton Dohrn, Fano Marine Centre, Viale Adriatico 1-N, 61032 Fano, Italy; michael.tangherlini@szn.it
2 Department of Materials, Environmental Sciences and Urban Planning, Polytechnic University of Marche, Via Brecce Bianche, 60131 Ancona, Italy; c.corinaldesi@staff.univpm.it
3 Institute of Anthropic Impacts and Sustainability in Marine Environment - National Research Council (IAS-CNR), Lungomare Cristoforo Colombo n. 4521 (ex complesso Roosevelt), Località Addaura, 90149 Palermo, Italy; francesca.ape@ias.cnr.it
4 Department of Integrated Marine Ecology, Stazione Zoologica Anton Dohrn, Rome; silvestro.greco@szn.it
5 Department of Integrated Marine Ecology, Stazione Zoologica Anton Dohrn, Sicily Marine Centre; teresa.romeo@szn.it; franco.andaloro@szn.it.
6 National Institute for Environmental Protection and Research, ISPRA, Italy; teresa.romeo@isprambiente.it
7 Department of Life and Environmental Sciences, Polytechnic University of Marche, Via Brecce Bianche, 60131 Ancona, Italy; r.danovaro@staff.univpm.it
* Correspondence: michael.tangherlini@szn.it;

Supplementary materials

Supplementary Figures 1-3

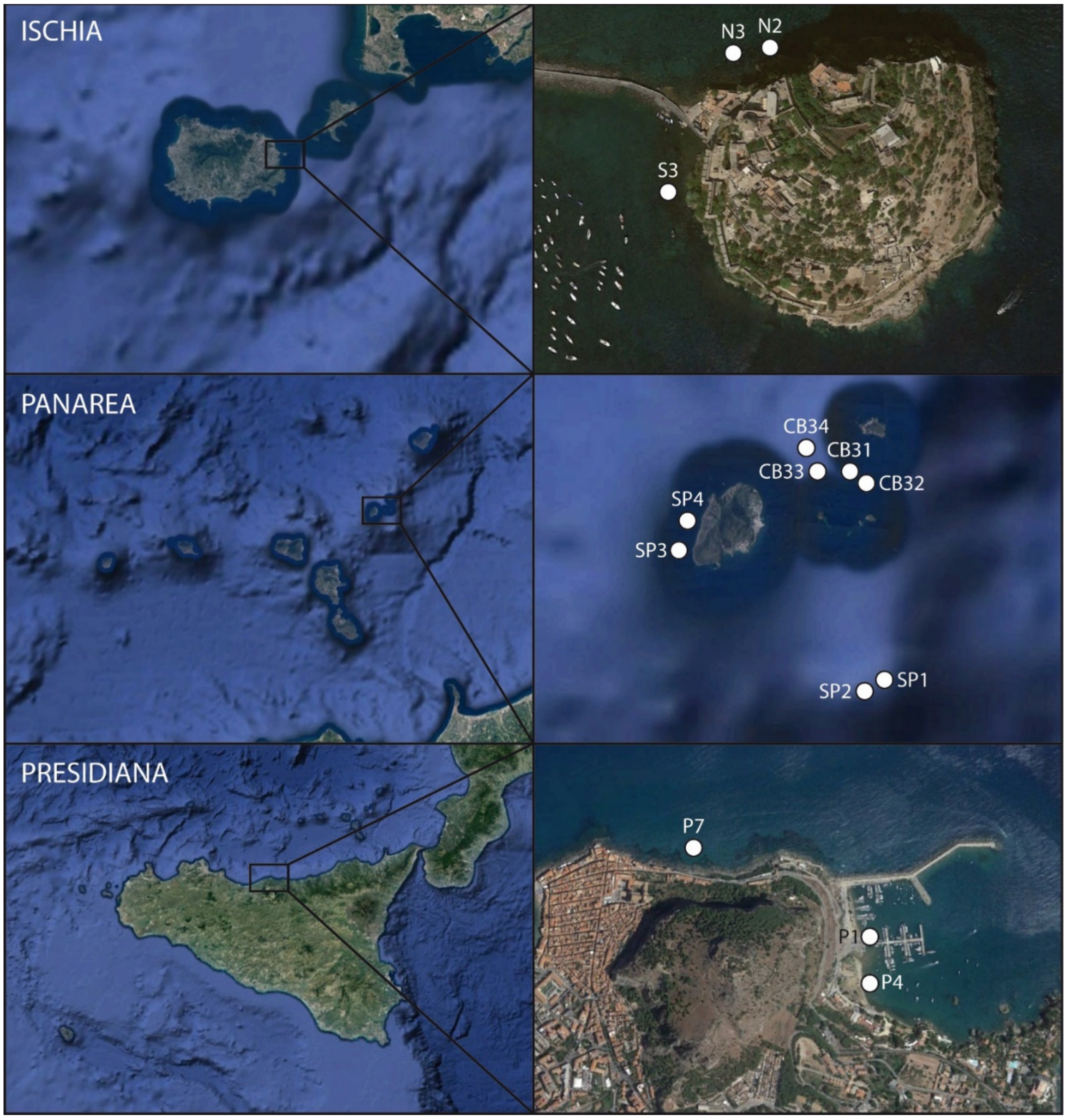

Supplementary Figure S1. Map of sampling areas and sampling sites.

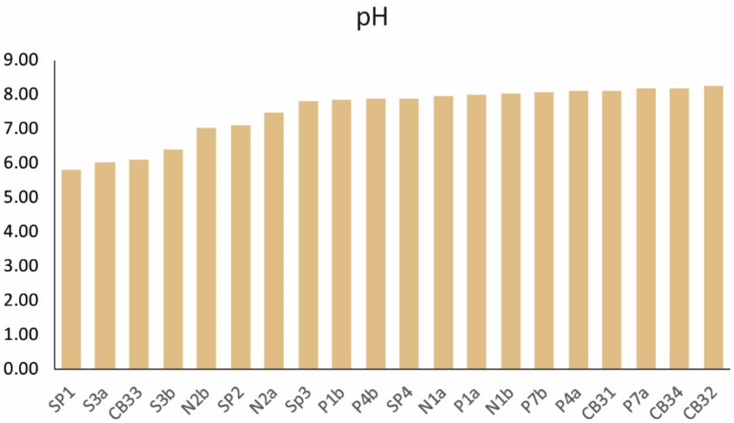

Scheme 2. Values of pH for all the sites investigated.

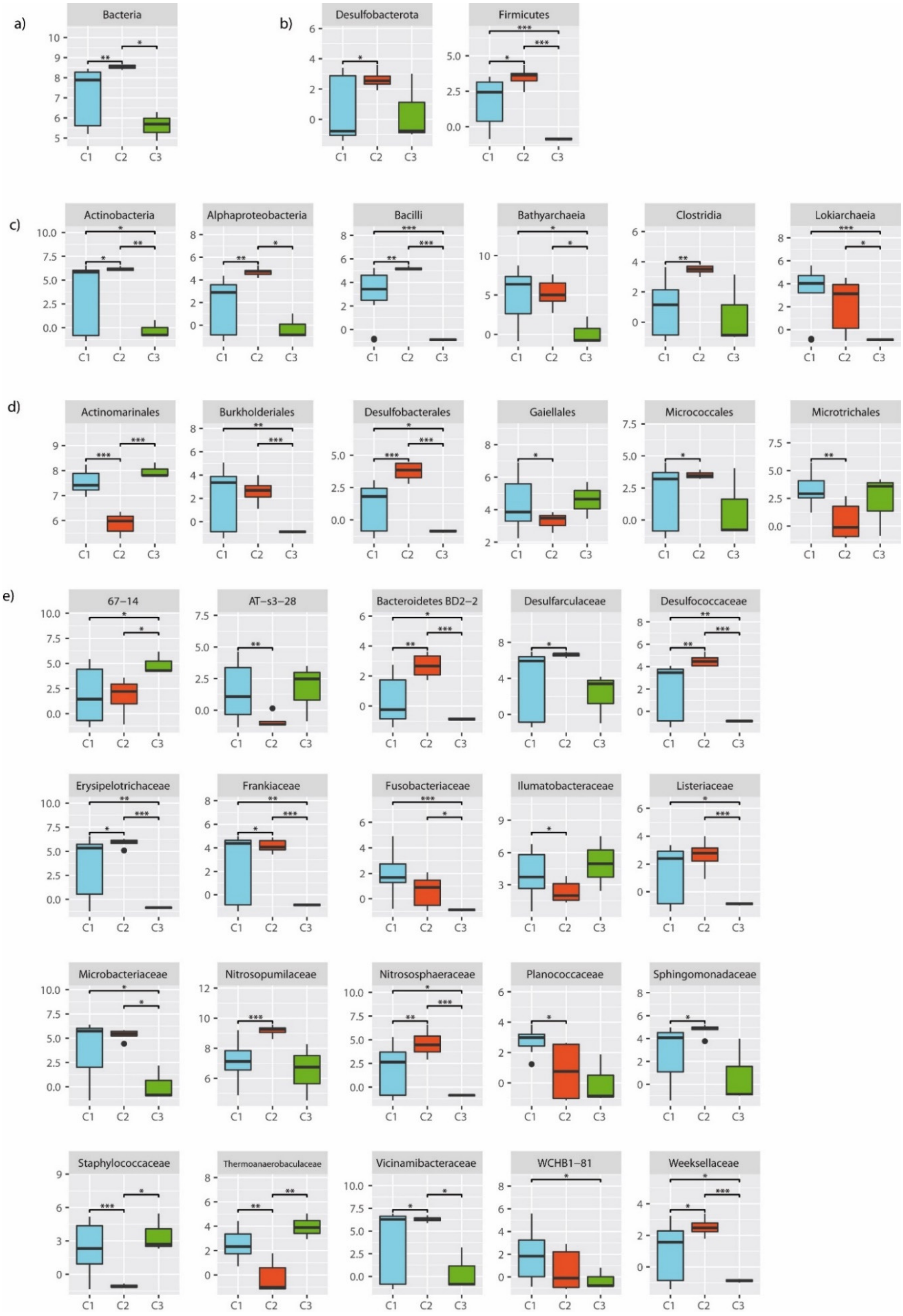

Scheme 3. CLR-transformed relative abundances of differentially abundant taxa classified at domain (A), phylum (B), class (C), order (D) and family (E) levels, with asterisks displaying significant differences between clusters (* for p-values < 0.05, ** for p-values < 0.01, *** for p-values < 0.001).
